# EHMTI-0184. Ictal adiponectin levels are modulated by pain severity and treatment response in episodic migraineurs

**DOI:** 10.1186/1129-2377-15-S1-E25

**Published:** 2014-09-18

**Authors:** NC Chai, B Gelaye, GE Tietjen, PD Dash, BA Gower, LW White, TN Ward, AI Scher, BL Peterlin

**Affiliations:** 1 Neurology, Johns Hopkins University School of Medicine, Baltimore, USA; 2 Epidemiology, Harvard School of Public Health, Boston, USA; 3 Neurology, University of Toledo, Toledo, USA; 4 Neurology, Johns Hopkins Community Physicians, Baltimore, USA; 5 Department of Nutrition Sciences, University of Alabama at Birmingham, Birmingham, USA; 6 Neurology, Dartmouth Hitchcock Medical Center, Lebanon, USA; 7 Preventive Medicine and Biometrics, Uniformed Services University, Bethesda, USA

## Introduction

Adiponection (ADP) and leptin (LEP) are adipokines with roles in inflammation.

## Aim

To assess ADP and LEP levels before and after acute abortive treatment in episodic migraineurs (EM).

## Methods

Peripheral blood specimens were collected from EM participants before and after acute abortive treatment with sumatriptan/naproxen sodium versus placebo.

## Results

A total of 34 participants (17 responders, 17 non-responders) were included. In all participants, for every 1 point increase in the HMW:T-ADP ratio, pain severity increased by 4.11 (CI: 0.44, 7.77; p=0.028). In responders (n=17), crude T-ADP levels were reduced at 30 min (11.49 ± 3.7; p=0.001), 60 min (11.54 ± 3.2; p=0.001) and 120 min (11.39 ± 3.7; p=0.001) after treatment as compared to onset (12.47 ± 3.6). In non-responders (n=17), crude T-ADP levels were unchanged after treatment. After adjustments, T-ADP levels remained decreased 30-120 min after treatment in responders; additionally, HMW-ADP, and the HMW:T-ADP ratio were decreased and LMW-ADP and the LMW:T-ADP ratio (coeff 0.04; CI: 0.01, 0.07; p=0.043) were increased 120 min after treatment in responders. In non-responders, the adjusted LMW-ADP (coef -0.45; CI: -0.77, -0.14; p=0.005) and the LMW:T-ADP ratio (coef -0.04; CI: -0.07, -0.01; p=0.018) were decreased at 60 min as well as at 120 min after treatment. Unadjusted and adjusted LEP levels were not modulated by changes in pain severity or treatment response.

## Conclusion

Adiponectin, but not leptin, is associated with pain severity and is modulated by treatment response in episodic migraineurs.

Conflict of interest.

